# Reprogramming of 3D genome structure underlying HSPC development in zebrafish

**DOI:** 10.1186/s13287-024-03798-x

**Published:** 2024-06-18

**Authors:** Min He, Xiaoli Li, Bingxiang Xu, Yinbo Lu, Jingyi Lai, Yiming Ling, Huakai Liu, Ziyang An, Wenqing Zhang, Feifei Li

**Affiliations:** 1 Division of Cell, Developmental and Integrative Biology, School of Medicine, South China University of Technology, Guangzhou, 510006 China; 2 Laboratory of Developmental Biology, Department of Cell Biology and Genetics, School of Basic Medical Sciences, Chongqing Medical University, Chongqing, 400016 China; 3 Key Laboratory of Hebei Province for Molecular Biophysics, Institute of Biophysics, School of Health Science & Biomedical Engineering, Hebei University of Technology, Tianjin, 300130 China; 4 Vehicle Engineering, School of Mechanical and Automotive Engineering, South China University of Technology, Guangzhou, 510000 China; 5 State Key Laboratory of Biocontrol, School of Life Sciences, Sun Yat-Sen University, Guangzhou, 510275 China

**Keywords:** HSPC, Chromatin conformation, Transcriptional regulation, Multi-omics dissection, PU.1

## Abstract

**Background:**

Development of hematopoietic stem and progenitor cells (HSPC) is a multi-staged complex process that conserved between zebrafish and mammals. Understanding the mechanism underlying HSPC development is a holy grail of hematopoietic biology, which is helpful for HSPC clinical application. Chromatin conformation plays important roles in transcriptional regulation and cell fate decision; however, its dynamic and role in HSPC development is poorly investigated.

**Methods:**

We performed chromatin structure and multi-omics dissection across different stages of HSPC developmental trajectory in zebrafish for the first time, including Hi-C, RNA-seq, ATAC-seq, H3K4me3 and H3K27ac ChIP-seq.

**Results:**

The chromatin organization of zebrafish HSPC resemble mammalian cells with similar hierarchical structure. We revealed the multi-scale reorganization of chromatin structure and its influence on transcriptional regulation and transition of cell fate during HSPC development. Nascent HSPC is featured by loose conformation with obscure structure at all layers. Notably, PU.1 was identified as a potential factor mediating formation of promoter-involved loops and regulating gene expression of HSPC.

**Conclusions:**

Our results provided a global view of chromatin structure dynamics associated with development of zebrafish HSPC and discovered key transcription factors involved in HSPC chromatin interactions, which will provide new insights into the epigenetic regulatory mechanisms underlying vertebrate HSPC fate decision.

**Supplementary Information:**

The online version contains supplementary material available at 10.1186/s13287-024-03798-x.

## Background

Understanding the regulatory mechanism underlying HSPC fate determination at different developmental stages is a primary goal of hematopoiesis biology. This is helpful in improving generation of functional HSPC in vitro. The HSPC development process is highly conserved between zebrafish and mammals and a series of important findings of HSPC ontology are based on zebrafish [1, 2]. For example, HSPC generation through endothelial-to-hematopoietic transition (EHT) is directly observed in zebrafish embryos [3]. There are three waves of hematopoiesis during zebrafish or mammalian development, with nascent HSPC arising from the ventral wall of dorsal aorta (DA) of zebrafish or aorta-gonad-mesonephros (AGM) region of mammals through the process of EHT, acquiring the ability of self-renewal and reconstruction of all blood lineages [4]. Then , this group of cells move to caudal hematopoietic tissue (CHT) of zebrafish or fetal liver of mammals to be fetal HSPC which can rapid expand and differentiate [5, 6]. Finally, these cells seed into kidney marrow (KM) of zebrafish or bone marrow of mammals, to become adult HSPC and support adult hematopoiesis [7].

Although significant achievements have been made to know this process, a comprehensive understanding of the dynamic regulatory mechanisms governing HSPC development is still lacking. Recent studies showed that despite the critical role of transcription factors (TFs), epigenetic modifications are also important in HSPC fate decision [8, 9]. Chromatin conformation is fundamental for transcriptional regulation via multiple mechanisms, from long-distance interactions between enhancers and promoters to higher-order chromosome compartments and topologically associated domains (TADs) that can act as transcription restrained units [10, 11]. Recent studies have shown 3D genome rearrangement participate in hematopoietic differentiation and disease [12–14]. Role of chromatin conformation on HSPC development have preliminary explored in mice [15, 16]. However, the development of HSPC is a multi-staged complex process , whether the regulatory role of 3D genome to HSPC development is conserved among vertebrate and what factors participate in regulation of HSPC development through 3D genome needs further investigation.

Here, we use zebrafish as a hematopoietic development model organism to investigate the dynamic changes in chromatin configuration during HSPC development. Multi-omics data, including sisHi-C, H3K27ac ChIP-seq, H3K4me3 ChIP-seq, ATAC-seq and RNA-seq were generated to comprehensively dissect 3D genome rearrangement and its relation to transcriptional changes and cell function of zebrafish HSPC. We found that 3D genome of zebrafish HSPCs are hierarchically organized, highly similar to those of mammalian cells. The development of zebrafish HSPC is accompanied by reprogramming of all layers of chromatin structure. In particular, the nascent HSPC is featured by more relaxed chromatin conformation. In addition, we identified a series of transcription factors potentially involved in mediating promoter-enhancer interactions and regulating HSPC development. Our study contributes to a deeper understanding of the epigenetic regulatory mechanisms underlying vertebrate HSPC development.

## Materials and methods

### Ethics approval and consent to participate

Title of the approved project: Reprogramming of 3D genome structure underlying HSPC development in zebrafish; Name of the institutional approval committee or unit: South China University of Technology Laboratory Animal Ethics Committee; Approval number: 2022105; Date of approval: January 15, 2022.

### Collection of zebrafish embryos

Zebrafish strains including Tubingen, Tg(CD41:GFP), Tg(gata1:dsRed), and Tg(CD41:GFP,gata1:dsRed) were raised under standard conditions (28.5 °C in system water). Zebrafish were raised, bred and staged according to the standard protocols. All experiments involving zebrafish were carried out in accordance with the guidelines set by the Institutional Animal Care and Use Committee of South China University of Technology. Collected zebrafish was euthanized by immersed in tricaine (MS222) for about 30 min. Our manuscript adheres to the ARRIVE guidelines 2.0 for the reporting of animal experiments.

### Flow cytometry

The trunk or tail region of zebrafish 36hpf or 3dpf embryos Tg(CD41:GFP, Gata1:DsRed) was cut for collection of nascent and fetal HSPC, respectively. Before the collection of trunks, embryos of 36 hpf were dechorionated using pronase. The trunk or tail region were ground, washed by grinding fluid and filtered using 100 μm cell-strainer. Then the dissociated cells were digested with dispase at 37 °C for 30 min into single cell suspension, followed by filtration using 40 μm cell-strainer. The kidney marrow of 3 mpf zebrafish were filtered to be single cell suspension using 40 μm cell-strainer after puffing well. Single cell suspension of different staged HSPCs were sorted and analyzed by flow cytometers MoFlo XDP (Beckman Coulter) in the purify model. The FACS data were analyzed with FlowJo software (v10, Tree star). Fluorescence markers for HSPC at different periods and regions were CD41+gata1–.

### In situ sisHi-C library preparation

The generation of in situ Hi-C library was performed as reported [17]. Briefly, cells sorted by FACS were taken to room temperature by mixing with an equal volume of PBS at 37 °C, and then fixed with freshly made 1% formaldehyde solution at room temperature for 10 min. 1.25 M glycine solution was added to a final concentration of 0.2 M for quenching the reaction. Cells were lysed in ice-cold Hi-C lysis buffer (10 mM Tris–HCl pH 8.0, 10 mM NaCl, 0.2% Igepal CA630, 1 × protease inhibitor cocktail) for 15 min. Pelleted nuclei were washed once with 1 × NEBuffer 2 and incubated in 0.5% sodium dodecyl sulfate (SDS) at 62 °C for 5 min. After incubating, water and Triton X-100 were added to quench the SDS. MboI restriction enzyme (NEB, R0147M) was added and chromatin was digested at 37 °C for 5 h. Biotin-14-dATP was used to mark the DNA ends followed by proximity ligation in intact nuclei. After crosslink reversal, DNA was sheared to a length of ∼300 bp with Covaris M220, then treated with the End Repair/dA-Tailing Module (NEB, E7442L) and Ligation Module (NEB, E7445L) following the operation manual. Biotin-labeled fragments were pulled down using Dynabeads MyOne Streptavidin C1 beads (Life technologies, 65001). The Hi-C libraries were amplified for 11–15 cycles with Q5 master mix (NEB, M0492L) following the operation manual. DNA was then purified with size selection. Libraries were then quantified and sequenced using NovaSeq platform (Illumina).

### ChIP-seq library preparation

ChIP-seq was conducted according to [18] with few modifications. The cells were cross-linked with a final concentration of 1% formaldehyde followed by quenching with glycine. Cells were lysed with lysis buffer (0.2% SDS;10 mM Tris -HCl, pH 8.0; 10 mM EDTA, pH 8.0; proteinase inhibitor cocktail) and sonicated to fragments about 300–500 bp (Bioruptor, Diagenode). Dynabeads Protein A was washed twice with ChIP Buffer (10 mM Tris–HCl pH7.5, 140 mM NaCl, 1 mM EDTA, 0.5 mM EGTA, 1% Triton X-100, 0.1% SDS, 0.1% Na-deoxycholate, Cocktail proteinase inhibitor) and was incubated with antibody at 4℃ for 2–3 h. The fragmented chromatin was transferred to the bead-antibody complex tubes and rotated at 4 °C overnight. The beads were washed once with low salt buffer (10 mM Tris–HCl pH7.5, 250 mM NaCl, 1 mM EDTA, 0.5 mM EGTA, 1% Triton X-100, 0.1% SDS, 0.1% Na-deoxycholate, Cocktail proteinase inhibitor) and twice with high salt buffer (10 mM Tris–HCl pH7.5, 500 mM NaCl, 1 mM EDTA, 0.5 mM EGTA, 1% Triton X-100, 0.1% SDS, 0.1% Na-deoxycholate, Cocktail proteinase inhibitor). After crosslink reversal, library was constructed as in-situ Hi-C. Antibodies used for H3K27ac and H3K4me3 are ab4729 and ab8580 (abcam), respectively.

### ATAC-seq library preparation

ATAC-seq was prepared as previously described with few modifications [19]. Briefly, 50,000 fresh cells were resuspended in 50 μl of ATAC-seq resuspension buffer (RSB; 10 mM Tris–HCl pH 7.4, 10 mM NaCl, and 3 mM MgCl2) containing 0.1% NP40, 0.1% Tween-20, and 0.01% digitonin and incubated on ice for 3 min. After lysis, 1 ml of ATAC-seq RSB containing 0.1% Tween-20 (without NP40 or digitonin) was used to wash nuclei. Nuclei were resuspended in 50 μl of transposition mix (10 μl 5XTTBL (Vazyme TD501), 5 μl TTE Mix V50, and 35 μl water) and pipetted up and down 20 times to mix. Transposition reactions were incubated at 37 °C for 30 min in a thermomixer. After the tagmentation, purify sample using the Ampure XP beads. The ATAC-seq library was amplified for 11 cycles of PCR with TAE mix (Vazyme TD501) following the manual. DNA was then purified with size selection, quantified and sequenced using an Illumina sequencing platform.

### RNA-seq library construction

The mRNA library was prepared using an optimized Smart-seq2 protocol [20]. Briefly, ∼10 ng of total RNA for each sample was utilized for first-strand cDNA reverse transcription in a 30 μl RT buffer containing SuperScript II RTase (100 U), RNase inhibitor (10 U), dNTP mix (10 mM each), SS III first-strand buffer (1×), DTT (5 mM), betaine (1 M), MgCl2 (6 mM) and TSO (1 μM). The RT reaction was performed at 90 min at 42 °C followed by 11 cycles of 2 min at 50 °C and 2 min at 42 °C for amplification and 15 min at 70 °C. The full-length cDNA was subsequently amplified through semi-suppressive PCR for 17 cycles in a buffer containing 30 μl of first-strand cDNA, 37.5 μl KAPA HiFi HotStart ReadyMix (1×), ISPCR primers (0.1 μM), and nuclease-free water. The amplified full-length DNA library was purified to get rid of < 500 bp fragments and other contaminants using AMPure XP beads (Beckman Coulter, A63881) following the manufacturer's protocol. 1 ng of purified DNA was exploited for library preparation using TruePrep DNA Library Prep Kit V2 for Illumina (Vazyme). DNA was then purified with size selection. Libraries were then quantified and sequenced using Illumina platform.

### Sequencing reads pre-processing and quality control

The quality of all libraries were evaluated by FastQC (http://www.bioinformatics.babraham. ac.uk/projects/fastqc/, v.0.11.9). Reads with mean quality score less than or equal to 30 are removed. Raw reads were trimmed and removed for adapter sequences by fatsp (v.0.23.2) with paired-end default parameters. Extremely short fragment with length less than or equal to 30 bp were also removed [21].

### ChIP-seq and ATAC-seq data processing

All ChIP-Seq and ATAC-seq reads were mapped to GRCz10 zebrafish genome by Bowtie2 (v.2.5.0) with very-sensitive configuration [22]. The uniquely aligned fragments with MAPQ ≥ 30 were extracted using SAMtools (v.1.9) [23]. Duplicates were removed by Picard tools MarkDuplicates (https://broadinstitute.github.io/picard/, v.2.27.4). ChIP-seq peaks were called using MACS2 with parameters “-f BMAPE” [24]. ATAC-seq peaks were also called using MACS2 callpeak command (v.2.2.5) with parameters “–nomodel –shift –100 –extsize 200 -q 0.05”. Peaks were first called in individual replicates. Then reads from different replicates were merged, and peak calling was performed with merged reads. Repeated peaks were then taken as those called from the merged reads that overlapped with those called in all replicates. Signal tracks were built using the bdgcmp sub-command of MACS2 with the fold enrichment over control (FE) mode. Active enhancers were defined as H3K27ac ChIP-seq peaks that not overlap with H3K4me3 peaks. To obtain differentially accessible regions, we merged peaks from all samples to obtain a non-redundant peak set. Read pair numbers for each non-redundant peak were calculated using HTseq (v0.8.0) and compared with DESeq2 [25, 26].

### RNA-seq data analysis

After filtering, high-quality reads were aligned to GRCz10 zebrafish genome using HISAT2 with default parameters [27]. FeatureCounts was used to quantify gene expression and obtain reads count. Fold changes in gene transcription levels were estimated using DESeq2 [28]. Enrichment analysis of gene function was performed in the Metascape platform (http://metascape.prg/gp/index.html). Active promoters were defined as 6 kb region centered on the transcription start site of genes with FPKM > 1.

### Hi-C data processing and visualization

HiC-Pro (v.3.1.0) was used for the processing of Hi-C data [29]. Only uniquely mapped read pairs with mapping quality no less than 10 were saved for further analysis, and dangling end reads, self-circled reads, and religated reads were all trimmed out. Non-duplicated reads were used to generate Hi-C contact matrices at the binning resolution of 10 kb, 50 kb and 100 kb. To validate the reproducibility of data, we calculated the GenomeDisco score between two libraries [30]. Contact heatmaps were generated with matrices at different resolutions by fanc (v.0.9.25) [31]. The p(s)-curves were calculated from genome distances of 20 kb to 50 Mb separated into 500 bins logarithmically. We applied the Von Neumann Entropy (VNE) approach to quantify the disorder of chromatin structure for 100-kb resolution intra-chromosomal matrices [32]. Hi-C matrix (M) was converted to correlation matrix C using corr (log2 [M]). Then, the eigenvalues (λi) of matrix C was obtained by eigen-decomposition and normalized with ${\uplambda i}=\frac{{\uplambda i}}{{\sum }_{j=1}^{n}{\uplambda j}}$. VNE was calculated as $-{\sum }_{i=1}^{n}{\uplambda iln}({\uplambda i})$.

Compartments were called d by analyzing the first eigenvector of the KR normalized contact maps at 100 kb resolution. The compartments with higher gene density were assigned as type A, while the compartments with lower gene density were assigned as type B. Compartment strength was calculated using AB/AA + BB. Saddle plots were calculated as previously described [33]. Hi-C matrix bins were sorted according to the PC1 values. Sorted frequencies were aggregated into 50 groups and averaged to obtain a compartmentalization saddle plot. Number of ATAC-seq peaks overlapped with compartments were analyzed by BEDtools with at least 1 bp shared [34].

TADs and TAD boundaries were identified at 50 kb resolution as described [35]. Shared TADs between different samples were defined as overlapping area larger than 75% for both samples. We calculated the standard deviations of the insulation score of each TAD boundary across three-cell stages and sorted boundaries by standard deviations. Then the top 1000 variable TAD boundaries were selected based on the ranking order. Clustering of boundaries was carried out using Pheatmap package in R.

Loops and interactions were detected with HiCCUPS in Juicer Tools at 10 kb resolution [36]. Enhancer or promoter involved loops were those with at least one anchor overlapped with enhancer regions (distal H3K27ac ChIP-seq peaks) or promoter regions (TSS ± 3 kb). Enhancer–promoter (E–P), promoter–promoter (P–P), and enhancer–enhancer (E–E) interactions were also identified. Shared loops were defined as loops with both anchors not shifting more than one bin. Aggregate peak analysis was processed with ‘apa’ in Juicer Tools, which generated aggregate heatmaps and average contact signals.

### TF motif analysis

Motifs were identified in H3K27ac ChIP-seq peaks located in loop anchors and the differential ATAC-seq peak regions using findMotifsGenome.pl in Homer [37]. The parameters were set as “-size given”. Only those motifs whose q-values smaller than 0.01 were treated as significantly enriched motifs.

### HPC7 analysis

ChIP-seq peaks of transcription factor, FPKM value of RNA-seq and loops identified from pcHi-C was downloaded from public data (GSE48086, GSE22178, E-MTAB-3954) [38–40]. Fraction of ChIP-seq peaks overlapping with loop anchors were compared with fractions of peaks overlapping with equal numbers of randomly chosen regions having same length with loop anchors. Genes were classified as PU.1 occupancy on both anchors if there is at least one loop connecting the gene promoter and having both anchors bound by PU.1. Genes with FPKM values ≥ 2 were considered as expressed.

## Results

### Adult HSPC of zebrafish exhibits hierarchical chromatin structure similar to mammalian cells

**Fig. 1:**
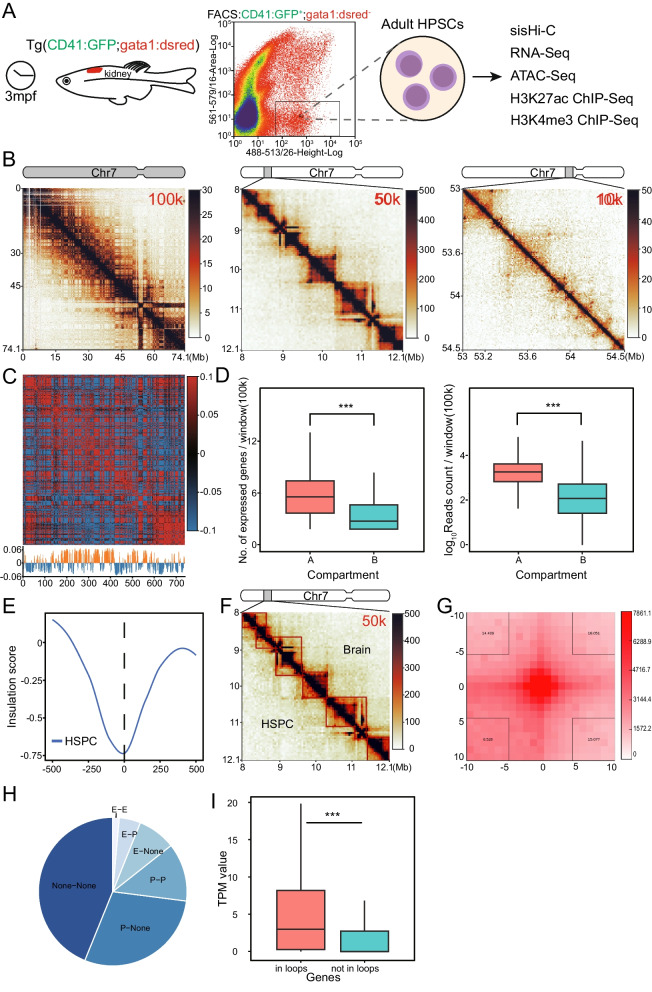
Characteristics of zebrafish HSPC 3D genome organization. **A** Schematic diagram of experimental design. **B** Hi-C contact matrix of chromosome 7 at 100 kb, 50 kb and 10 kb resolution are showed as example. **C** Chromatin compartmentalization of chromosome 7. The autocorrelation matrices and the first eigenvector profiles are shown. In the first eigenvector, compartment B is colored as blue and A as orange. **D** Boxplot showing the distribution of gene density and RNA-seq reads density in the A/B compartment. **E** Genome-wide insulation score profiles around TAD boundaries. **F** TADs detected in 8-12 Mb region of chromosome 7 in both HSPC and brain are shown as an example. **G** Aggregate loop plots showing the strength of interactions between HSPC loop anchors. **H** The distribution of identified interactions on functional elements. E, enhancer; P, promoter; None, neither enhancer nor promoter. **I** Distribution of transcript per million (TPM) expression value of genes involved and not involved in loops. ***p < 0.001

In order to reveal the chromatin structure of zebrafish HSPCs for the first time, we performed four replicates of sisHi-C on FACS sorted cd41+gata1-adult HSPCs from three-month-old transgenic zebrafish Tg(cd41:GFP gata1:DsRed). RNA-seq, ATAC-seq, H3K4me3 ChIP-seq and H3K27ac ChIP-seq were also conducted to illustrate the characteristic of HSPC chromatin folding (Fig. 1A). A total of 172842225 valid pairs were obtained from the four Hi-C replicates. GenomeDisco analysis showed replication score of any two replicates are higher than 0.85 at both 50 kb and 100 kb resolution, so we combined the four replicates in the following analysis (Fig. S1A). The Hi-C contact map of zebrafish adult HSPC showed canonical hierarchical chromatin organization at different resolutions, including compartments, TAD and loops, similar to mammalian cells (Figs. 1B and S2).

At the compartment level, about half of the genomic regions were assigned as A and B compartments, respectively (Fig. 1C). We found that A compartments contained more expressed genes (*p* < 2.22e−16, wilcox.test) and the expression level of encompassed genes was also higher compared with B compartments (*p* < 2.22e−16, wilcox.test, Fig. 1D). A compartment also showed more enrichment of H3K27ac, H3K4me3 ChIP-seq peaks and ATAC-seq open regions comparing with B compartments (*p* < 2.22e−16 and < 2.22e−16, wilcox.test, respectively, Fig. S1B). These results showed that A compartments are more active in zebrafish HSPC. At 50 kb resolution, a total of 1643 TADs with a median size of 800 kb were identified. The accuracy of called TADs was verified by the strongest insulation of aggregated boundaries (Fig. 1E). Similar to mammalian cells, the TAD boundaries of zebrafish HSPC enriched for transcribed TSS (Fig. S1C). In order to illustrate the conservation of TAD structures between tissues, we analyzed publicly available Hi-C data of zebrafish brain (GSE134055) [41] and detected 1595 TADs. Of these, 1175 TADs are shared between brain and HSPC (Fig. 1F). The overlap ratio almost approach that of biological replicates of HSPC (Fig. S1D). Although most majority of TADs are conserved, there are 420 brain- and 468 adult HSPC-specific TAD boundaries. We analyzed the function of genes located in tissue-specific TAD boundaries and found that enriched pathways are related to the function of specific tissues. For example, phospholipid metabolic process, which is important for brain function, is most enriched in brain specific boundaries (Fig. S1E) [42]. Finally, at the loop level, a total of 4189 loops were detected in adult HSPC. The aggregated peak analysis (APA) showed high confidence of identified loops (Fig. 1G). We identified distal enhancers genome-widely taking advantage of H3K27ac and H3K4me3 ChIP-seq data, and loops were assigned to functional elements. We found that most majority of identified loops connect enhancer (E) or promoter (P) (Fig. 1H). In addition, expression of genes involved in loops are significantly higher than genes not connected by loops, implying the functionality of detected loops (*p* < 2.22e−16, wilcox.test, Fig. 1l).

In summary, we conducted the first investigation into the chromatin conformation of zebrafish HSPC and discovered a hierarchical organization with similar features as mammalian cells.

### Dispersed chromatin structure in zebrafish nascent HSPC

**Fig. 2:**
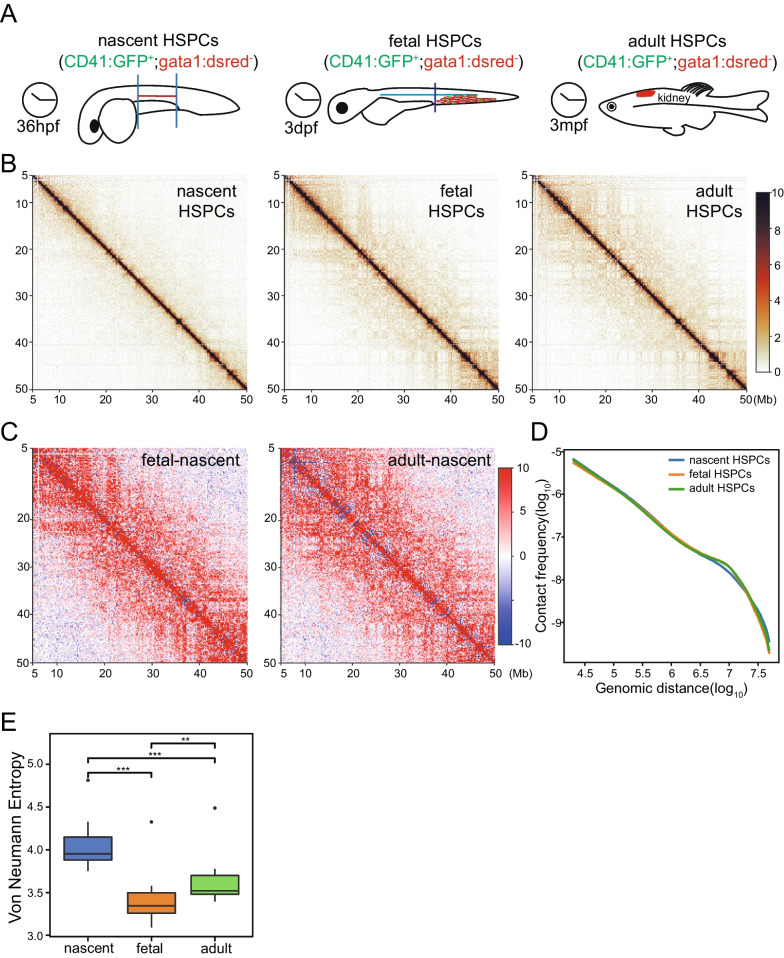
Global reorganization of chromatin structure during zebrafish HSPC development. **A** Schematic representation of chromatin conformation detection for nascent HSPCs in the AGM region at 36hpf and fetal HSPCs in the CHT region at 3dpf. **B** A 45 Mb region of chromosome 7 is shown with 50-kb resolution as an example of the contact maps during the process of HSPC development. **C** Contact heatmaps of fetal and adult HSPC were subtracted by that of nascent HSPC for the same region as B. **D** Contact frequency decay curves at different stages of HSPC development. **E** Quantification of the intra-chromosomal disorder in chromatin structure using Von Neumann Entropy (VNE)

We want to reveal the reprogramming of chromatin structure and its contribution to the development of HSPC. Nascent HSPCs from the AGM region at 36 hpf, as well as fetal HSPCs from the CHT region at 3 dpf were collected and performed sisHi-C for at least two replicates (Fig. 2A). A total of 26350389 and 118555641 valid pairs were obtained for nascent and fetal HSPC, respectively (Table S1). High reproducibility of Hi-C experiments was validated by a median GenomeDISCO score of nearly 0.8 for all replicates (Fig. S3A). To make the Hi-C data of different stages comparable, we downsampled the pooled valid pairs to the number of nascent HSPC.

Hi-C contact maps are more similar between fetal HSPC and adult HSPC, but substantially different from nascent HSPC (Fig. 2B). Firstly, median GenomoDisco score of Hi-C contact maps were 0.844 and 0.904 comparing fetal HSPC with nascent and adult HSPC, respectively (Fig. S3B). Secondly, interactions in nascent HSPC are concentrated near diagonal area, while more long-range interactions spanning dozens of megabases were observed in fetal and adult HSPC visually. This trend was obvious when subtracting contact matrix of fetal and adult HSPC by the matrix of nascent stage (Fig. 2C). Thirdly, contact frequency decay curves of fetal and adult HSPC are more similar (Fig. 2D). Jensen–Shannon divergence (JSD) was 0.0017 and 0.0012 comparing fetal HSPC with nascent and adult HSPC, respectively. Especially, decay curves showed depletion of contacts at distance of ~ 10 Mb and slower decrease at distance of ~ 30 Mb in nascent HSPC, which reminded a more relaxed chromatin organization [12]. In addition, higher proportion of inter-chromosomal interactions in nascent HSPC also indicated loose structure (Table S1).

Chromatin structure, transcriptome and chromatin accessibility indicate more relaxed structure of nascent HSPC. Chromosomal level Von Neumann Entropy (VNE) index was calculated to quantify chromatin disorder (Fig. 2E), and the result showed that the entropy of nascent HSPC was significantly higher than that of fetal and adult HSPC, indicating more disordered organization. We also compared transcriptome and chromatin accessibility of the three stages (CRA001858) [43]. Clustering analysis showed that transcriptome changes are more pronounced from nascent to fetal stages (Fig. S3C). Importantly, the number of significantly downregulated genes is more than twice of upregulated genes from nascent to fetal HSPC (Fig. S3D). Chromatin openness exhibit similar feature with more pronounced difference between nascent and fetal HSPC, and nearly two folds of regions become closed than that become accessible (Fig. S3E and F). These results showed that transcription and chromatin availability were consistent with chromatin structure and support more relaxed conformation of nascent HSPC.

In conclusion, gene transcription, chromatin accessibility and 3D genome structure were dynamic changed during zebrafish HSPC development, especially from nascent to fetal stages. Chromatin of nascent HSPC was more relaxed.

### Coordination of compartments and chromatin accessibility in transcriptional regulation

**Fig. 3:**
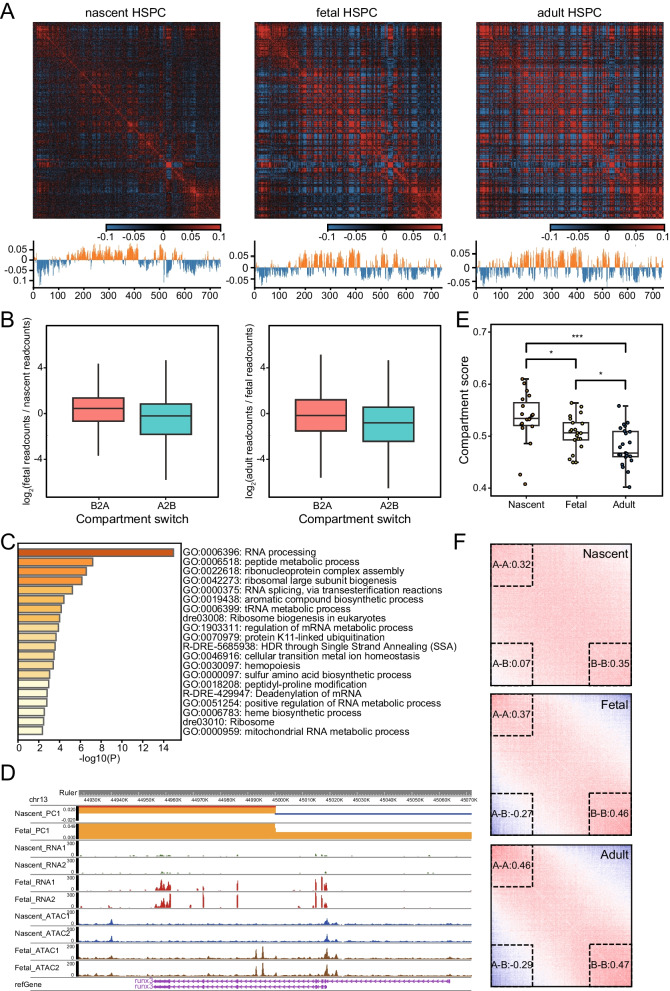
Compartment reprogramming associated with expression changes during HSPC development. **A** Chromatin compartmentalization at the three developmental stages. Chromosome 7 is shown as an example as in Fig. 1C but with down-sampled data. The autocorrelation matrices and the first eigenvector profiles are shown. **B** Expression changes of genes located within compartment switching regions. **C** Enriched pathways of genes located in B2A switch region and upregulated from nascent to fetal HSPC. **D** Switching of compartment assignment near runx3 gene is shown by first eigenvector profiles. ATAC-Seq and RNA-Seq signal for both replicates are displayed in the rest of the tracks. **E** The chromosome-wise compartment scores at each developmental stage. **F** Compartmentalization saddle plots of different stages during HSPC development. Numbers represent relative interaction strength between compartments

We subsequently explored the reprogramming of the 3D genome at the sub-chromosome level. Using contact maps at 100 kb resolution, we identified 47.7–49% of the genome as accessible A compartments (Fig. 3A). These regions exhibit higher gene density and transcriptional activity compared with B compartments at all stages (Fig. S4A). We found that switching of A/B compartments affect gene expression and cell function. About 18% and 12% of genomic regions undergo compartment changes from nascent to fetal HSPC and from fetal to adult HSPC, respectively (Fig. S4B). Genes contained within regions changing from B to A compartment tend to be upregulated, while those contained in A to B tend to be downregulated (Fig. 3B). In addition, function of these genes was correlated with HSPC stage-specific characteristics. For example, Genes switched from B to A compartment and showed transcriptional upregulation from nascent to fetal HSPC enriched in pathways of ‘RNA processing’ and ‘ribosome biogenesis’ (Fig. 3C), while from fetal to adult HSPC, ‘lipid biosynthetic’ and ‘phagocytosis’ related pathways are enriched (Fig. S4C), in accordance with the rapid proliferation of fetal HSPC and adaptive immunity of adult HSPC [44, 45].

To further determine whether changes in compartmentalization occur coincidently with local changes in chromatin accessibility, we compared compartment assignment with ATAC-seq data. A compartment had higher chromatin accessibility than B compartments in all stages (Fig. S4D), and the stage-specific ATAC peaks mainly occurred in stage-restricted compartment A regions (Fig. S4E and F). These indicate the coordination of large-scale compartment and local chromatin accessibility. We detected some important regulatory factors switched compartments and changed accessibility during HSPC development. For example, runx3 was previously reported to contribute to the maintenance of HSCs in fetal liver, but play no role at the onset of definitive hematopoiesis [46]. It located in B compartment in nascent HSPC with low gene expression and chromatin accessibility, while changed to A compartment with higher expression and accessibility in fetal HSPC (Fig. 3D).

Next, we compared the strength of compartmentalization at the three HSPC stages. Visual inspection showed that the Pearson autocorrelation matrix for nascent HSPC appears less obvious (Fig. 3A). Compartment scores (AB/AA + BB) were calculated and the result showed that score of nascent HSPC is significantly higher than fetal HSPC, indicating weaker compartmentalization (*p* = 0.015, wilcox.test, Fig. 3E). Compartment score of fetal HSPC was also significantly higher than adult HSPC, implying the gradually obvious compartmentalization during HSPC development (*p* = 0.033, wilcox.test, Fig. 3E). Saddle plot showed the same trend with increasing separation of A and B compartments, especially from nascent to fetal stage (Fig. 3F). The above results indicated that reorganization of compartmental structures and chromatin accessibility were associated with changes of gene expression and HSPC function during zebrafish HSPC developmental.

### TADs largely kept stable but much weaker at nascent stage during HSPC development

**Fig. 4:**
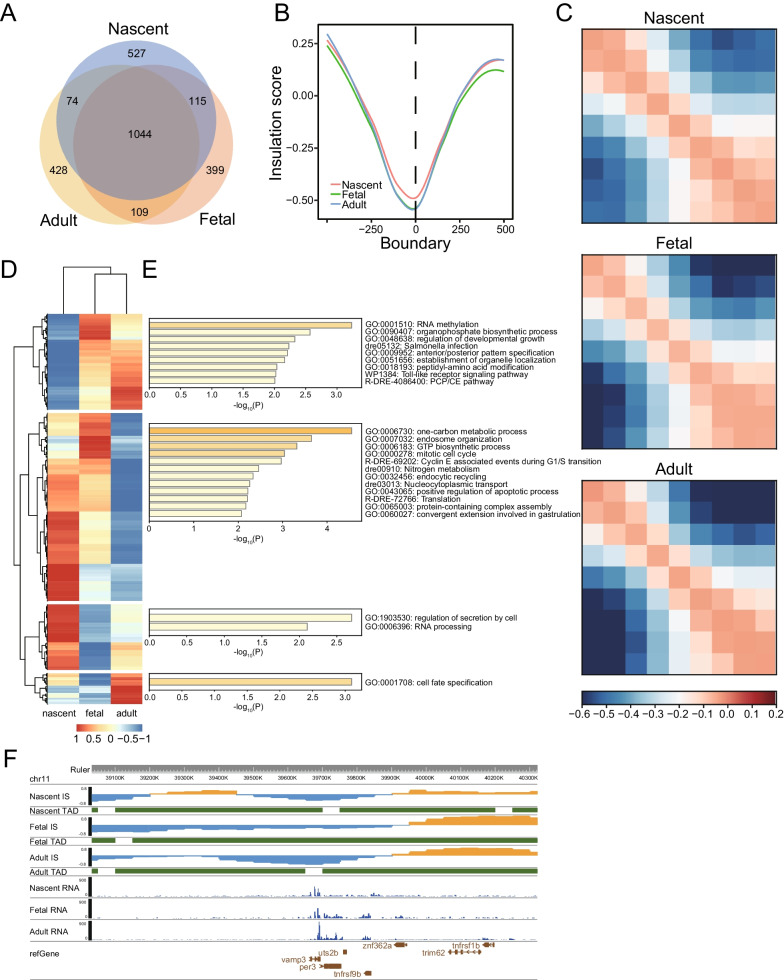
TAD structure kept relatively stable but much weaker in zebrafish nascent HSPC. **A** Venn graphs showing the overlap of TADs between different stages of HSPC. **B** Genome-wide insulation score profiles around TAD boundaries. **C** Observed/expected (O/E) aggregate plot of interaction profile centered on TAD boundaries for the three stages of HSPC. The upper left and lower right corners represent the strength of intra-TAD interactions, while the upper right and lower left corners represent the strength of inter-TAD interactions. **D** Heatmap showing the clustering of top 1000 variable TAD boundaries based on insulation score. Each row was one TAD boundary. **E** Gene ontology analysis for genes located within TAD boundary clusters. **F** Insulation score, TAD structure as well as RNA-seq signal was shown near vamp3 gene for fetal and adult HSPC

At a finer scale, a total of 1760 and 1667 TADs were identified with median length of about 750 kb and 800 kb in nascent and fetal HSPC, respectively. Accuracy of TADs were verified by the lowest IS at aggregated TAD boundaries. The position of TADs remained largely unshifted during HSPC development (Fig. S5A). There are 1044 TADs were shared by all three stages, which is similar to that observed between adult HSPC and brain (Figs. 4A, S1D). In addition, when comparing two consecutive stages, about 60% TAD boundaries remained stable and not shifting more than one bin. This proportion is similar to that observed between two biological replicates (Fig. S5B).

Then, we compared TAD strength of the three stages of HSPC and found strength are much weaker in nascent HSPC. Insulation score (IS) of TAD boundaries in fetal HSPC is comparable with adult HSPC, with mean IS of − 0.530 and − 0.527, respectively (*p* = 0.92, paired t-test, Fig. 4B). However, IS of nascent HSPC is much higher than fetal HSPC, indicating weaker separation between TADs (mean IS = − 0.478, *p* = 0.07, paired t-test, Fig. 4B). Compared with nascent HSPC, fetal and adult HSPC showed increased intra-TAD interactions and decreased inter-TAD interactions in the stacked interaction profile centered on TAD boundaries (Fig. 4C). In order to dissect the influence of changed TAD boundaries, we identified the top 1000 highly variable boundaries based on IS among the nearly 4000 bins that were used as TAD boundaries in any stage. The 1000 boundaries can be clustered into four categories, which correspond to the three developmental stages specific boundaries and common boundaries of nascent and fetal stages (Fig. 4D). Gene ontology analysis showed that genes encompassed in these stage-biased boundaries are associated with cell function of specific stage (Fig. 4E). For example, cluster 2 showed active endocytic related pathways, consistent with adult HSPC function of adaptive immunity. We observed some stage-specific functional genes located in variable boundaries. Insulation score near VAMP3, which is associated with endocytosis and facilitates membrane fusion [47, 48], is decreased in adult HSPC compared with fetal HSPC, accompanied by new TAD boundary establishment and VAMP3 upregulation in adult HSPC (Fig. 4F).

### PU.1 potentially mediate promoter-involved looping interactions in HSPC

**Fig. 5:**
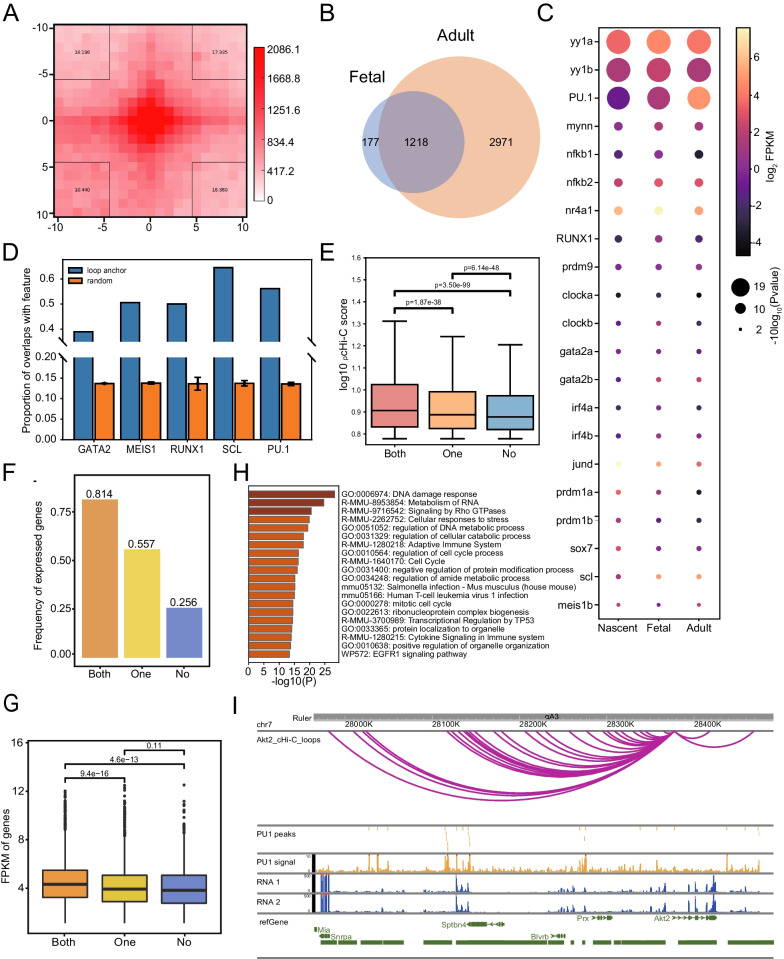
PU.1 mediate promoter involved chromatin looping in adult HSPC. **A** Aggregate loop plots showing the strength of interactions between fetal HSPC loop anchors. **B** Overlap of loops between fetal and adult HSPC. **C** Bubble plots showing gene expression and TF motif enrichment identified at H3K27ac peak region in adult HSPC loop anchors. Enriched p-value was calculated by HOMER. **D** Bar chart showing the overlap proportion of the TF-binding peaks with the loop anchors (blue) or background random selected regions (orange) for HPC7 cell. **E** Interaction score for different groups of loops based on PU.1 occupancy on loop anchors. **F** Proportion of expressed genes for different groups of genes based on PU.1 occupancy on loops connecting gene promoter. **G** Transcriptional level for expressed genes in different groups as in E. **H** Gene ontology analysis for genes having loops connecting gene promoter with both anchors occupied by PU.1. **I** Promoter capture Hi-C loops, PU.1 peaks and signal, as well as RNA-seq signal was present near Akt2 gene for HPC7 cell line

We compared the finer scale loop structure of fetal and adult HSPC, due to the relatively limited valid pairs impaired loop detection of nascent HSPCs. A total of 1395 loops were identified in fetal HSPC at the resolution of 10 kb (Fig. 5A). Of these, 1218 loops were shared (with both anchors shifting no more than one bin) between fetal and adult HSPC (Fig. 5B). Further quantitative analysis showed the alteration of loops is actually minor. We plot the APA profile of the 2971 adult HSPC-specific loops using nascent and fetal contact matrix, and found that interactions between anchors of these loops are also higher than neighboring regions in both nascent and fetal stages (Fig. S6A). In addition, cosine similarity of contact frequencies of these 2971 loops were as high as 0.87 and 0.93 when comparing nascent with fetal HSPC and fetal with adult HSPC, respectively. These observations indicated that adult HSPC specific contacts actually had been concentrated in nascent and fetal stages, which is further strengthened at adult HSPC.

We attempt to identify transcriptional factors with role in mediating chromatin interactions and transcriptional activation in zebrafish HSPC. Focusing on transcriptional regulatory loops, we did motif analysis on H3K27ac ChIP-seq peak regions located in loop anchors of adult HSPC. Result showed that in addition to ETS-domain transcription factor family, such as PU.1, 31 other TFs were identified whose motifs are enriched (*p* < 0.01). As our results showed loop interactions of adult HSPC were already existed in nascent and fetal stages, so we paid attention to TFs that are expressed in all three stages. The homologs of 15 TFs in zebrafish are expressed in all three stages based on RNA-seq data (Fig. 5C). Several TFs with known function in HSPC development were identified, such as TAL1, RUNX1, GATA1 and PU.1 [38, 49]. The most enriched TFs are YY1 and PU1. In addition, expression of PU.1 was gradually increased, which may contribute the enhanced interaction strength of adult HSPC. However, YY1 transcriptional level kept relatively stable during HSPC development (Fig. 5C). These results indicated that PU.1 may mediate loop interactions in zebrafish HSPC.

In order to clarify the potential of identified TFs mediating loop structures and regulate transcription, we utilized a common blood stem/progenitor cell model HPC-7, which has ChIP-seq data of 5 (PU.1, GATA2, RUNX1, SCL and MEIS1) out of the 15 candidate TFs as well as high-resolution promoter-capture Hi-C (pcHi-C) and RNA-seq data (GSE48086, GSE22178, E-MTAB-3954) [38–40]. We calculated the frequency of the 5 TF peaks that overlap with the interacting fragments identified by pcHi-C, and compared it with randomly picked noninteracting control regions. All of the 5 TFs showed significant enrichment at interacting regions indicating their potential role in genomic looping (Fig. 5D). We also calculated number of loops that have specific TF binding, and found PU.1 was most frequently present on loop anchor (Fig. S6B). Nearly half of promoter-involved loops have PU.1 binding on both or one anchor. To investigate function of PU.1 in mediating chromatin loops, we directly compared the pcHi-C score of loops with both anchors, one anchor or no anchor having PU.1 binding. Although all these interactions were called as loops, interaction score are significantly higher for loops with both anchors having PU.1 binding than loops with one anchor binding. The difference is also significant for loops with one PU.1 binding than no binding (Fig. 5E). The result indicated that PU.1 may mediate or at least strengthen the looping interactions. We further analyzed influence of PU.1 binding to transcription. Genes were classified into three groups based on PU.1 occupancy on both anchors, one anchor or no anchor of loops connecting gene promoters. We found more proportion of genes are expressed and the gene expression level is significantly higher in genes with both anchors binding by PU.1 than genes with one anchor binding by PU.1 (Fig. 5F and G). This difference is also obvious when comparing genes with one anchor binding by PU.1 and no anchor binding. Gene ontology analysis showed that genes with two loop anchors occupied by PU.1 are enriched in ‘cell cycle’ and ‘immune system’ related pathways, in accordance with the rapid proliferation and multipotent hematopoietic differentiation of HPC7 cells (Fig. 5H). Several well-known genes important for cell cycle and immune reaction having PU.1 binding on both anchors of loops connecting its promoter, such as Akt2 and Cdk2 (Figs. 5I and S6C) [50].

Taken together, our results implied that PU.1 may mediate 3D genome looping interactions and potentially regulate gene expression of HSPC.

## Discussion

By integrating multi-omics datasets generated from 3D genome structure, transcriptome, chromatin accessibility as well as histone modification, this study reports the structural dynamic of multi-layered 3D genome and its contribution to shape HSPC ontogeny in zebrafish for the first time. In particular, PU.1 was detected and verified by public data that potentially mediates chromatin loop formation and regulates gene expression as well as HSPC characteristics.

We found that chromatin of zebrafish HSPC are organized into hierarchic structure with similar feature as mammalian cells. During development of HSPC, the obscure 3D genome structure in nascent HPSC was strengthened in fetal and adult HSPC at all layers, including compartments, TADs and loops. Integrating with studies in mouse, the reprogramming of chromatin structure during HSPC development have commonalities and specificity between species. Murine fetal and adult HSPCs preserved large-scale compartments and TADs structure, while intra-TAD interactions are more dynamic [15]. This is in accordance with our results, which showed highly similar contact frequency decay curves as well as conserved position and comparable strength for both compartments and TADs between zebrafish fetal and adult HSPC. The loop structure was more variable, with fewer loops and weaker strength in fetal HSPC compared with adult HSPC. However, although murine nascent HSPC did not show impaired chromatin structure [16], our results in zebrafish nascent HSPC illustrated more relaxed chromatin organization and compromised strength for compartmentalization and TADs. The disordered structure in zebrafish nascent HSPC was supported by changes in chromatin accessibility, gene expression as well as the chromatin entropy. This may underlie the substantial molecular and phenotypical differences between nascent and fetal HSPCs showed by previous studies [5, 51]. In addition, in agreement with 3D structure in early mammalian embryos is obscure but gradually enhanced during development [52], the relatively relaxed structure highlights a highly plastic state at the early stages of HSPC development and may be important for transitions from endothelial to hematopoietic properties.

The ETS-family transcription factor PU.1 is a key regulator of hematopoiesis. PU.1 is activated in HSPC and is expressed in mast cells, B cells, granulocytes, and macrophages but is switched off in T cells. Previous studies illustrate that PU.1 play crucial roles in the development of both myeloid and lymphoid lineages as well as lymphoid-primed multipotent progenitors [53–55]. For HSPC, PU.1 is important for maintenance or expansion of HSPC number in murine fetal liver [56], and for homing and long-term engraftment in the bone marrow [57]. In addition, bone marrow HSCs disrupted with PU.1 in situ could not maintain hematopoiesis and were outcompeted by normal HSCs. PU.1 also limits hematopoietic stem cell expansion and prevents exhaustion of adult HSPC [58]. These results illustrate multiple functions of PU.1 in HSPC development, maintenance and differentiation [59]. We provided evidence that PU.1 may regulate HSPC gene expression through mediating chromatin loops. Some studies have illustrated PU.1 can function as loop mediator at specific loci or genes [60, 61]. As far as we know, for the first time, our results proposed the genome-widely structural function of PU.1 in mediating enhancer-promoter interactions in HSPC. In addition, the evidence from murine HPC7 support the conservation of PU.1 structural roles between species. One recent study in murine HSPC highlighted RUNX1 engaged in chromatin interactions and promoted hematopoiesis. Interestingly, RUNX1 and PU.1 were shown to have physical interactions [62], and the relationship of these two proteins as well as other interaction partners in mediating chromatin interactions in HSPC needs further investigation. In addition, more direct evidence, such as PU.1 HiChIP [63] or ChIP-loop [64], is needed to validate the function of PU.1 in mediating chromatin interactions. The rarity of in vivo HSPC especially in nascent and fetal stage is a limitation, and the analysis maybe achieved with the development of low-input detection methods in the future.

In summary, zebrafish is a widely used model system for HSPC research, and to our knowledge, this is the first research studying the feature of chromatin conformation and its dynamic during HSPC development in zebrafish. We revealed contribution of 3D genome reprogramming to transcriptional regulation and HSPC fate transition. Zebrafish nascent HSPC is featured by the loose structure that is not observed in mouse, which emphasized the species specificity. In addition, runx1-engaged enhancer-promoter interactions were found to promote hematopoiesis during the emergency of nascent HSPC in mouse. Our study paid more attention to the regulatory role of 3D genome to the development of HSPC and revealed PU.1 mediating chromatin loop formation and potentially regulating gene expression during HSPC development. We believe research from different species will expand our understanding of the regulation mechanism of HSPC fate determination.

## Conclusions

Our findings demonstrate that the chromatin organization of zebrafish HSPC resemble mammalian cells with similar hierarchical structure. Nascent HSPC is featured by loose conformation with obscure structure at all layers. Notably, PU.1 was identified as a potential factor mediating the formation of promoter-involved loops and regulating gene expression of HSPC. Our results provided a global view of chromatin structure dynamics associated with development of zebrafish HSPC and discovered key transcription factor involved in HSPC chromatin interactions, which will provide new insights into the epigenetic regulatory mechanisms underlying vertebrate HSPC fate decision.

### Supplementary Information

Supplementary Material 1: Figure S1. Chromatin conformation of zebrafish adult HSPC. (A) GenomeDisco scores showing the reproducibility of the Hi-C libraries. (B) Distribution of H3K27ac, H3K4me3 ChIP-seq and ATAC-seq peak density in the A/B compartments. (C) Boxplot showing the proportion of TAD boundaries overlapped with gene transcriptional start site (TSS) and compared with randomly selected regions having same length as TAD boundaries. (D) Venn graphs showing the overlap of TADs between adult HSPC and brain as well as between biological replicates of HSPC. (E) The enriched biological processes of genes located in tissue-specific TAD boundaries.

Supplementary Material 2: Figure S2. Interaction heatmap of all chromosomes at 100kb, 50kb and 10kb resolutions.

Supplementary Material 3: Figure S3. Global transcriptional and chromatin conformation changes during HSPC development. (A) GenomeDisco scores showing the reproducibility of the Hi-C libraries. (B) GenomeDisco scores of consecutive stages during HSPC development. (C) Clustering analysis of gene expression data for different stages of HSPC samples. (D) Volcano plot of differentially expressed genes (Fold change ≥ 2, Padj < 0.05) for consecutive stages during HSPC development. (E) Clustering analysis of ATAC-seq signal on accessible peak regions. (F) Volcano plot of differentially accessible regions based on ATAC-seq (Fold change ≥ 1.5, Padj < 0.05) for consecutive stages during HSPC development.

Supplementary Material 4: Figure S4. Changes of compartmentalization during zebrafish HSPC development. (A) Boxplot showing the distribution of transcript per million (TPM) expression value of genes in the A/B compartment for nascent and fetal HSPC. (B) Proportions of genome regions which switched compartments. (C) Enriched pathways of genes located in B2A switch region and upregulated from fetal to adult HSPC. (D) Distribution of ATAC-seq peak density in the A/B compartments for all developmental stages. (E) Overlap of differentially regulated ATAC-seq peaks from nascent to fetal HSPC with stage-specific compartment A regions. (F) The same as E, but from fetal to adult HSPC.

Supplementary Material 5: Figure S5. TADs kept relatively stable during zebrafish HSPC development. (A) Illustration of stable TAD structure taking chr1: 7.8-11.1Mb as an example. (B) Bar plot showing overlap of TAD boundaries between biological replicates and successive developmental stages.

Supplementary Material 6: Figure S6. Candidate transcription factors mediating chromatin looping interactions in HSPC. (A) Aggregate loop plots showing contact frequencies of adult HSPC-specific loops in nascent and fetal HSPC cells. (B) Frequency of HPC7 loops occupied by each transcription factor. (C) Same as 5H, but near Cdk2 gene.

Supplementary Material 1:

## Data Availability

All raw sequencing data generated in this study have been submitted to GEO under accession number GSE262185-GSE262188, and GSE262738. The data also deposited in the Genome Sequence Archive in the National Genomics Data Center [65], Beijing Institute of Genomics (BIG), Chinese Academy of Sciences (https://ngdc.cncb.ac.cn/) under accession numbers CRA014187 and can be viewed at https://ngdc.cncb.ac.cn/gsa/s/aPxmb6T2.

## Abbreviations

HPSC: Hematopoietic stem and progenitor cells
EHT: Endothelial-to-hematopoietic transition
DA: Dorsal aorta
AGM: Aorta-gonad-mesonephros
CHT: Caudal hematopoietic tissue
KM: Kidney marrow
TFs: Transcription factors
TADs: Topological associated domains
APA: Aggregated peak analysis
E: Enhancer
P: Promoter
JSD: Jensen–Shannon divergence
VNE: Von Neumann entropy
IS: Insulation score
