# Supplementary figures and images for: Reprogramming of 3D genome structure underlying HSPC development in zebrafish
Source: Stem Cell Res Ther. 2024 Jun 18;15:172. doi: 10.1186/s13287-024-03798-x (PMC11184745; doi:10.1186/s13287-024-03798-x)

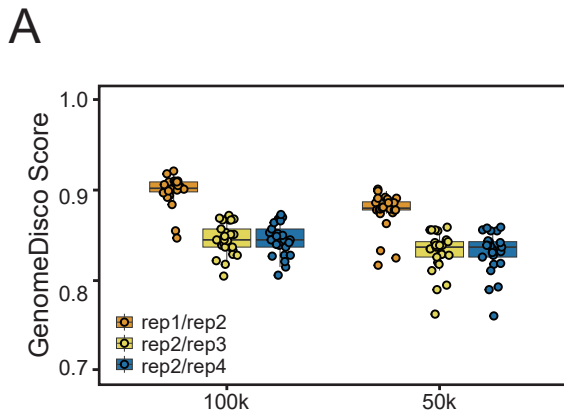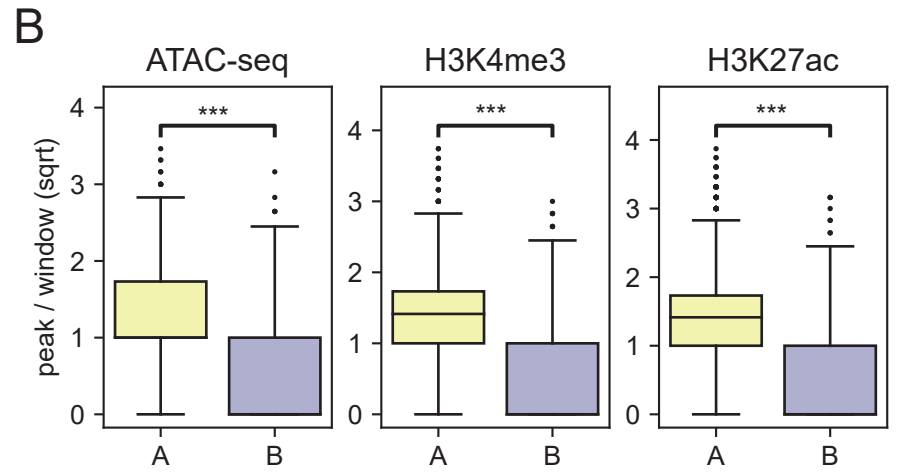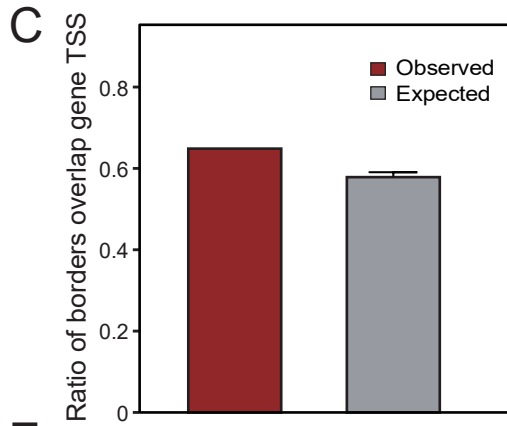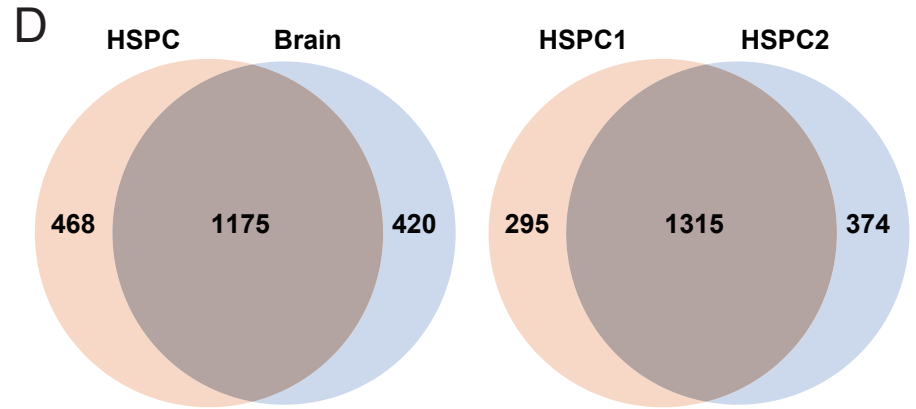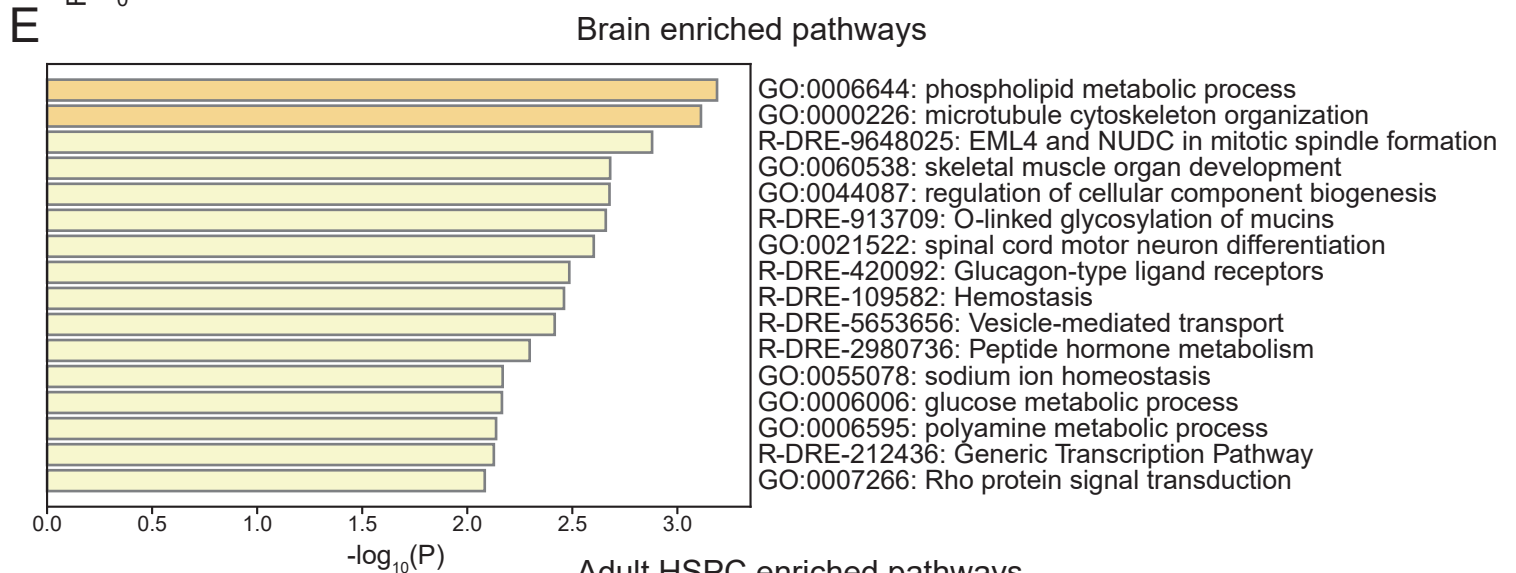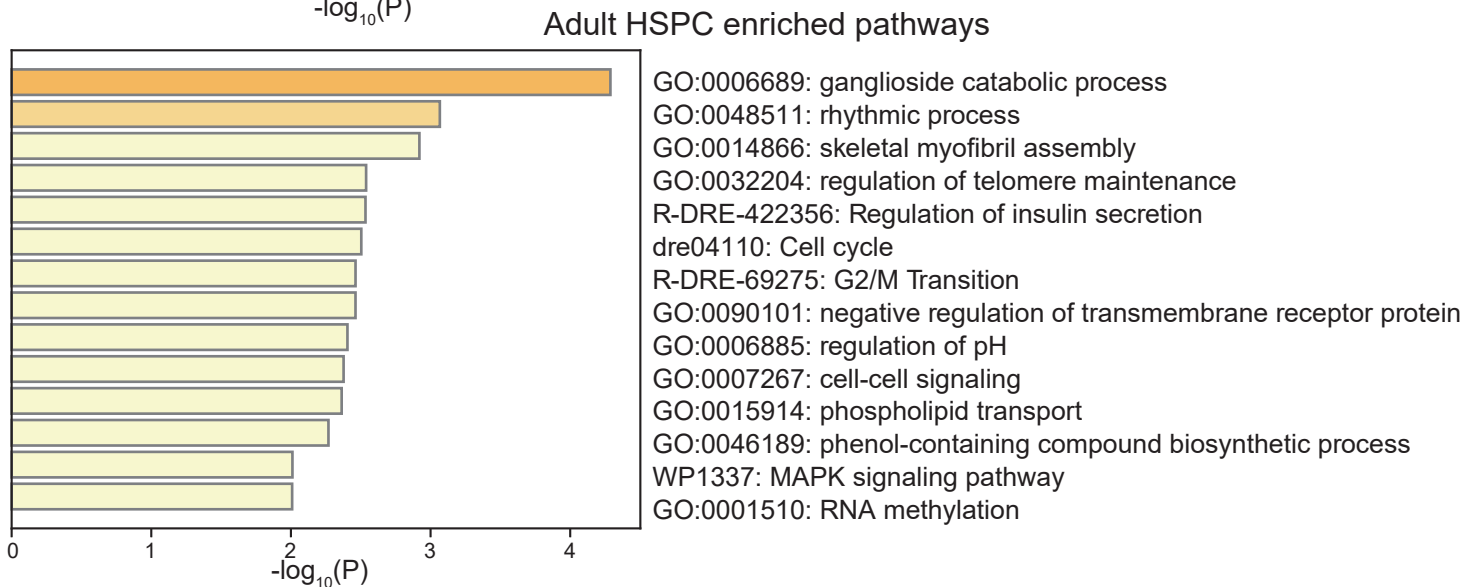

Supplement: Supplementary file 1 — Supplementary Material 1: Figure S1. Chromatin conformation of zebrafish adult HSPC. (A) GenomeDisco scores showing the reproducibility of the Hi-C libraries. (B) Distribution of H3K27ac, H3K4me3 ChIP-seq and ATAC-seq peak density in the A/B compartments. (C) Boxplot showing the proportion of TAD boundaries overlapped with gene transcriptional start site (TSS) and compared with randomly selected regions having same length as TAD boundaries. (D) Venn graphs showing the overlap of TADs between adult HSPC and brain as well as between biological replicates of HSPC. (E) The enriched biological processes of genes located in tissue-specific TAD boundaries. [file 13287_2024_3798_MOESM1_ESM.pdf]

**Figure S2**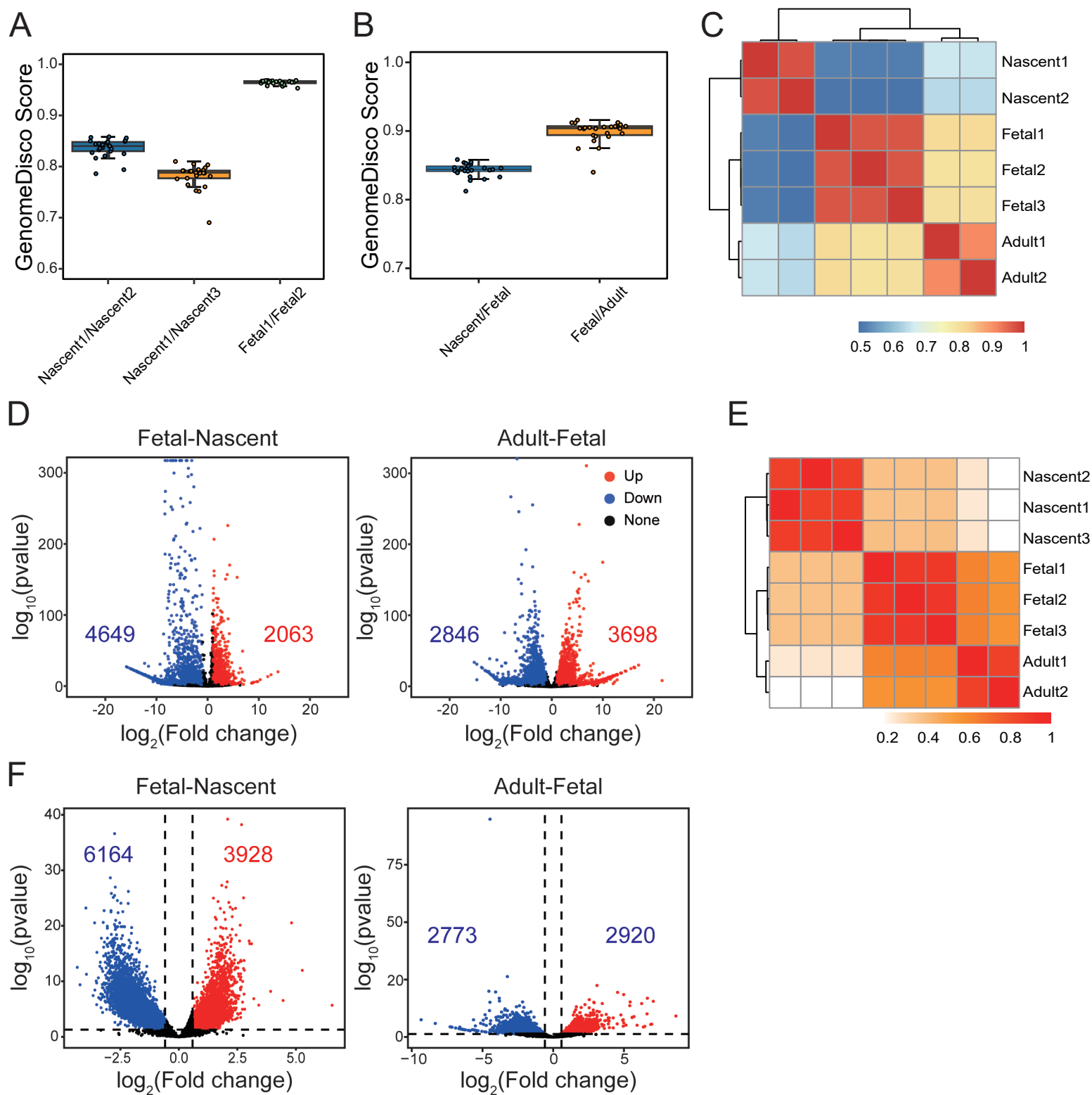

Supplement: Supplementary file 3 — Supplementary Material 3: Figure S3. Global transcriptional and chromatin conformation changes during HSPC development. (A) GenomeDisco scores showing the reproducibility of the Hi-C libraries. (B) GenomeDisco scores of consecutive stages during HSPC development. (C) Clustering analysis of gene expression data for different stages of HSPC samples. (D) Volcano plot of differentially expressed genes (Fold change ≥ 2, Padj < 0.05) for consecutive stages during HSPC development. (E) Clustering analysis of ATAC-seq signal on accessible peak regions. (F) Volcano plot of differentially accessible regions based on ATAC-seq (Fold change ≥ 1.5, Padj < 0.05) for consecutive stages during HSPC development. [file 13287_2024_3798_MOESM3_ESM.pdf]

Figure S3

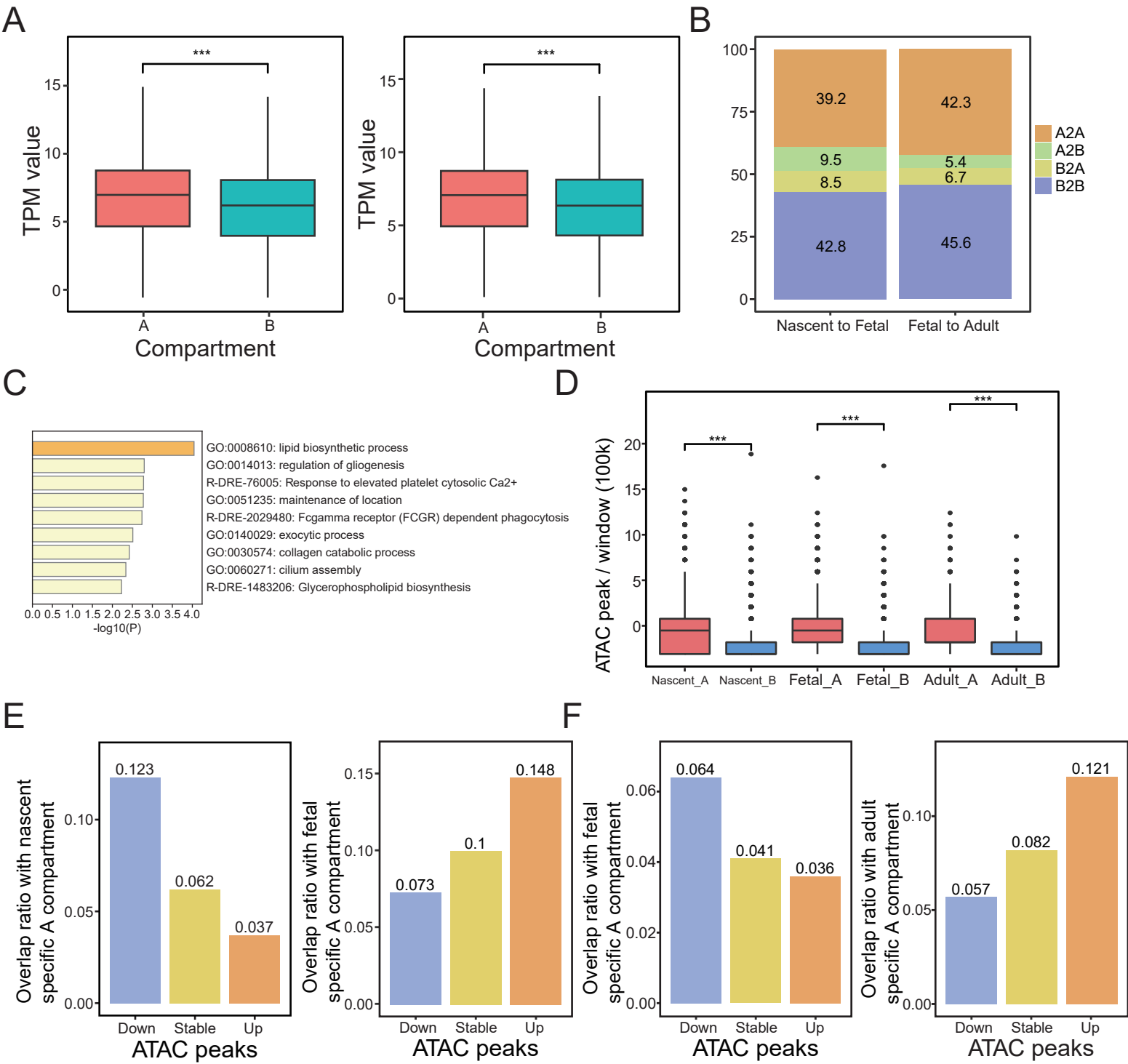

Supplement: Supplementary file 4 — Supplementary Material 4: Figure S4. Changes of compartmentalization during zebrafish HSPC development. (A) Boxplot showing the distribution of transcript per million (TPM) expression value of genes in the A/B compartment for nascent and fetal HSPC. (B) Proportions of genome regions which switched compartments. (C) Enriched pathways of genes located in B2A switch region and upregulated from fetal to adult HSPC. (D) Distribution of ATAC-seq peak density in the A/B compartments for all developmental stages. (E) Overlap of differentially regulated ATAC-seq peaks from nascent to fetal HSPC with stage-specific compartment A regions. (F) The same as E, but from fetal to adult HSPC. [file 13287_2024_3798_MOESM4_ESM.pdf]

Figure S4

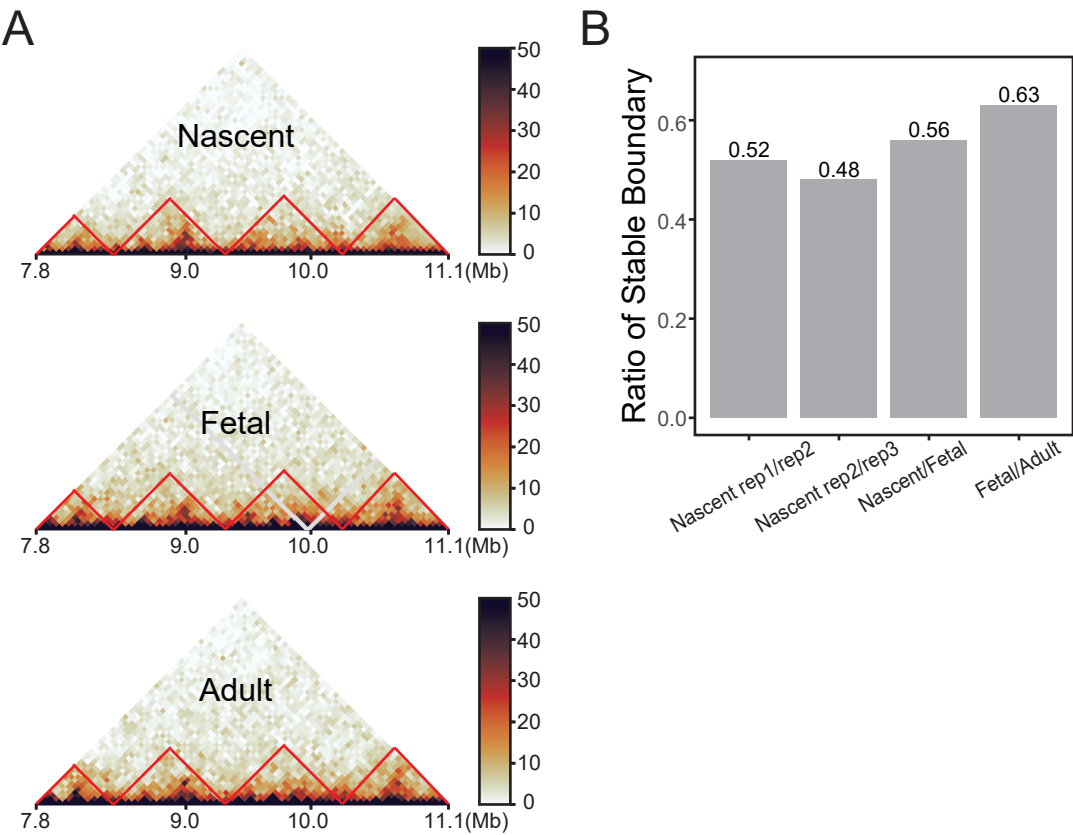

Supplement: Supplementary file 5 — Supplementary Material 5: Figure S5. TADs kept relatively stable during zebrafish HSPC development. (A) Illustration of stable TAD structure taking chr1: 7.8-11.1Mb as an example. (B) Bar plot showing overlap of TAD boundaries between biological replicates and successive developmental stages. [file 13287_2024_3798_MOESM5_ESM.pdf]

### Figure S5

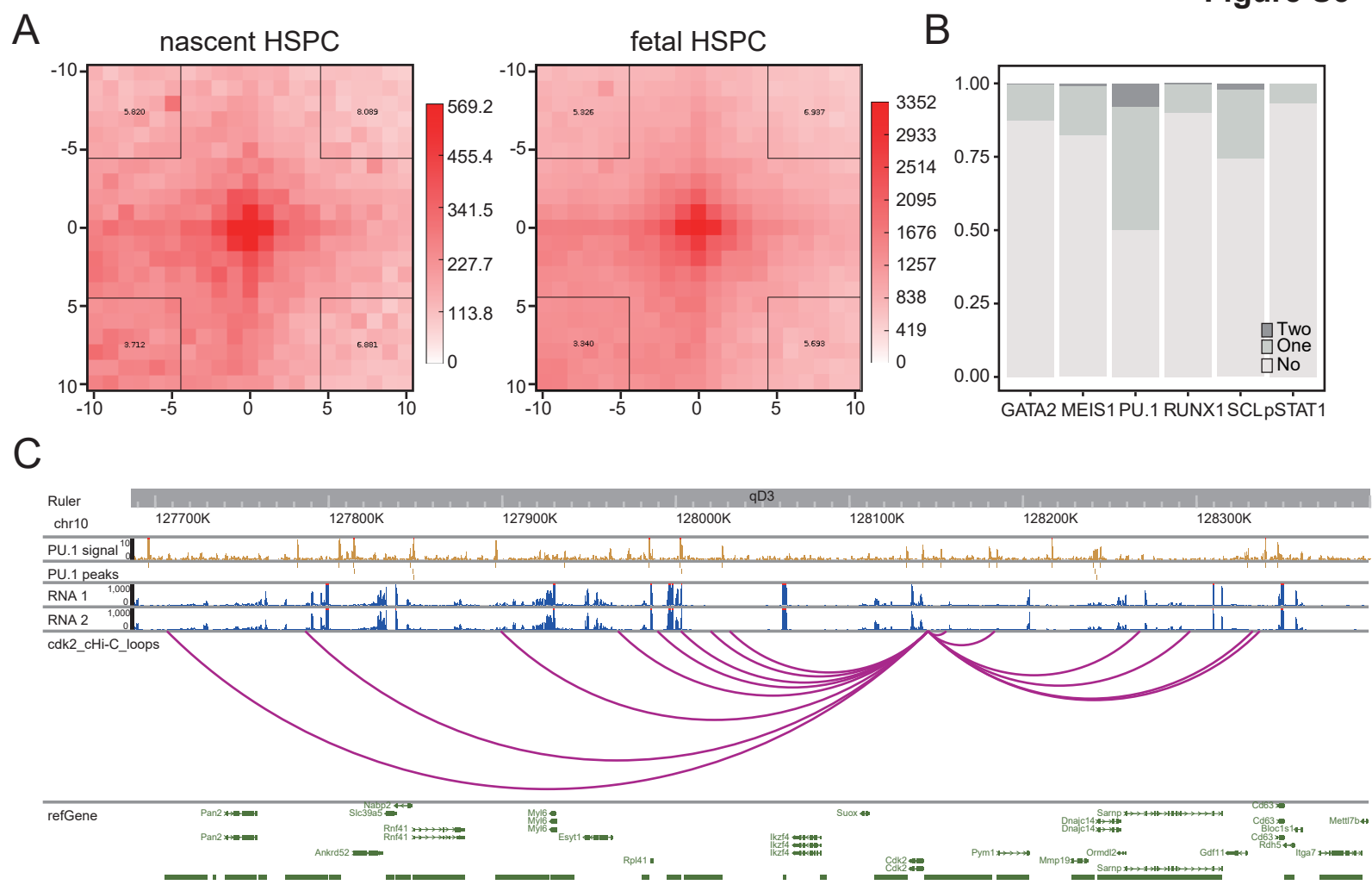

Supplement: Supplementary file 6 — Supplementary Material 6: Figure S6. Candidate transcription factors mediating chromatin looping interactions in HSPC. (A) Aggregate loop plots showing contact frequencies of adult HSPC-specific loops in nascent and fetal HSPC cells. (B) Frequency of HPC7 loops occupied by each transcription factor. (C) Same as 5H, but near Cdk2 gene. [file 13287_2024_3798_MOESM6_ESM.pdf]
