# Supplementary material for: Reprogramming of 3D genome structure underlying HSPC development in zebrafish
Source: Stem Cell Res Ther. 2024 Jun 18;15:172. doi: 10.1186/s13287-024-03798-x (PMC11184745; doi:10.1186/s13287-024-03798-x)
Supplement: Supplementary file 2 — Supplementary Material 2: Figure S2. Interaction heatmap of all chromosomes at 100kb, 50kb and 10kb resolutions. [file 13287_2024_3798_MOESM2_ESM.pdf]

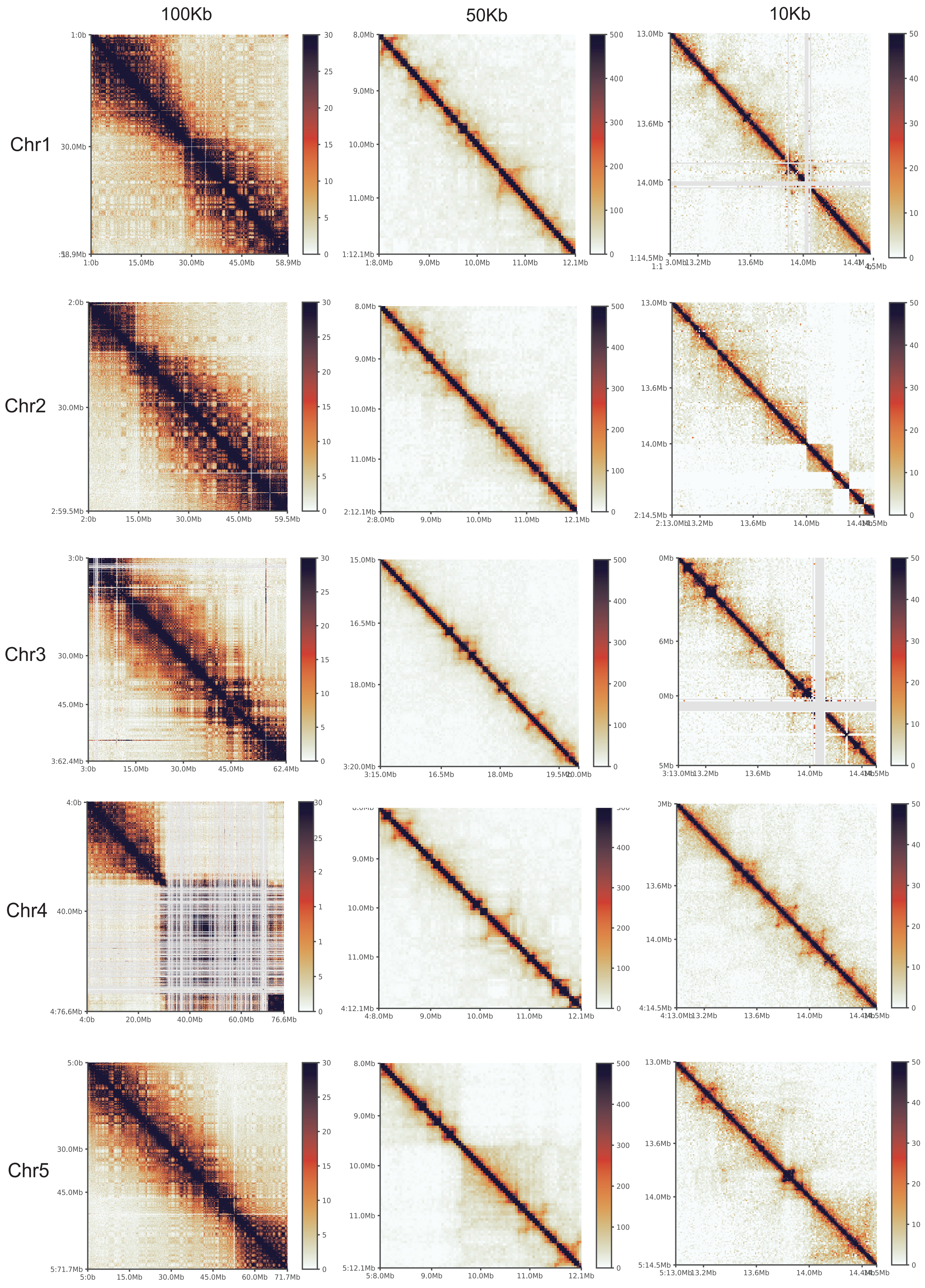
100Kb
50Kb
10Kb
Chr1
Chr2
Chr3
Chr4
Chr5

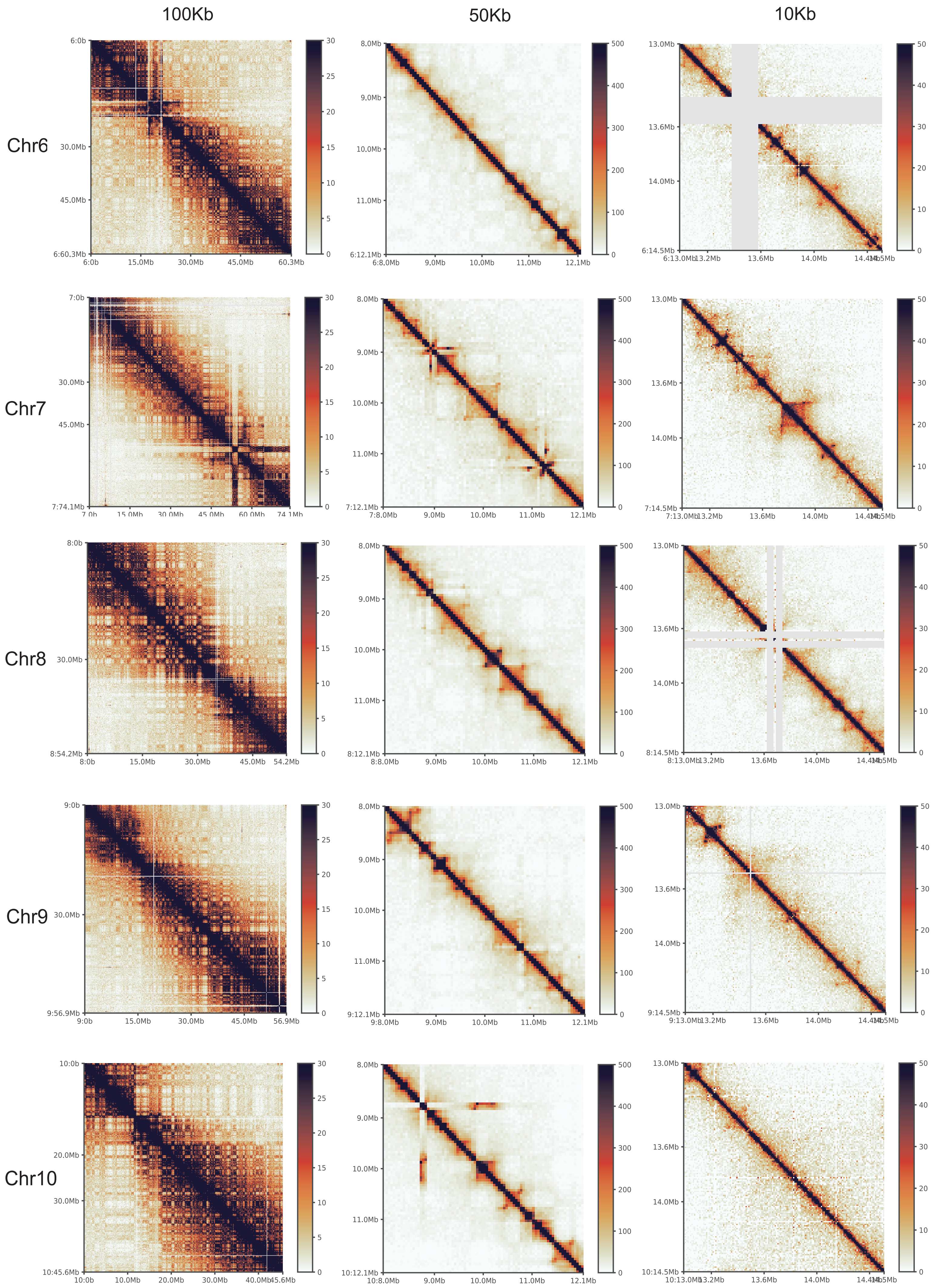
100Kb
50Kb
10Kb
Chr6
Chr7
Chr8
Chr9
Chr10

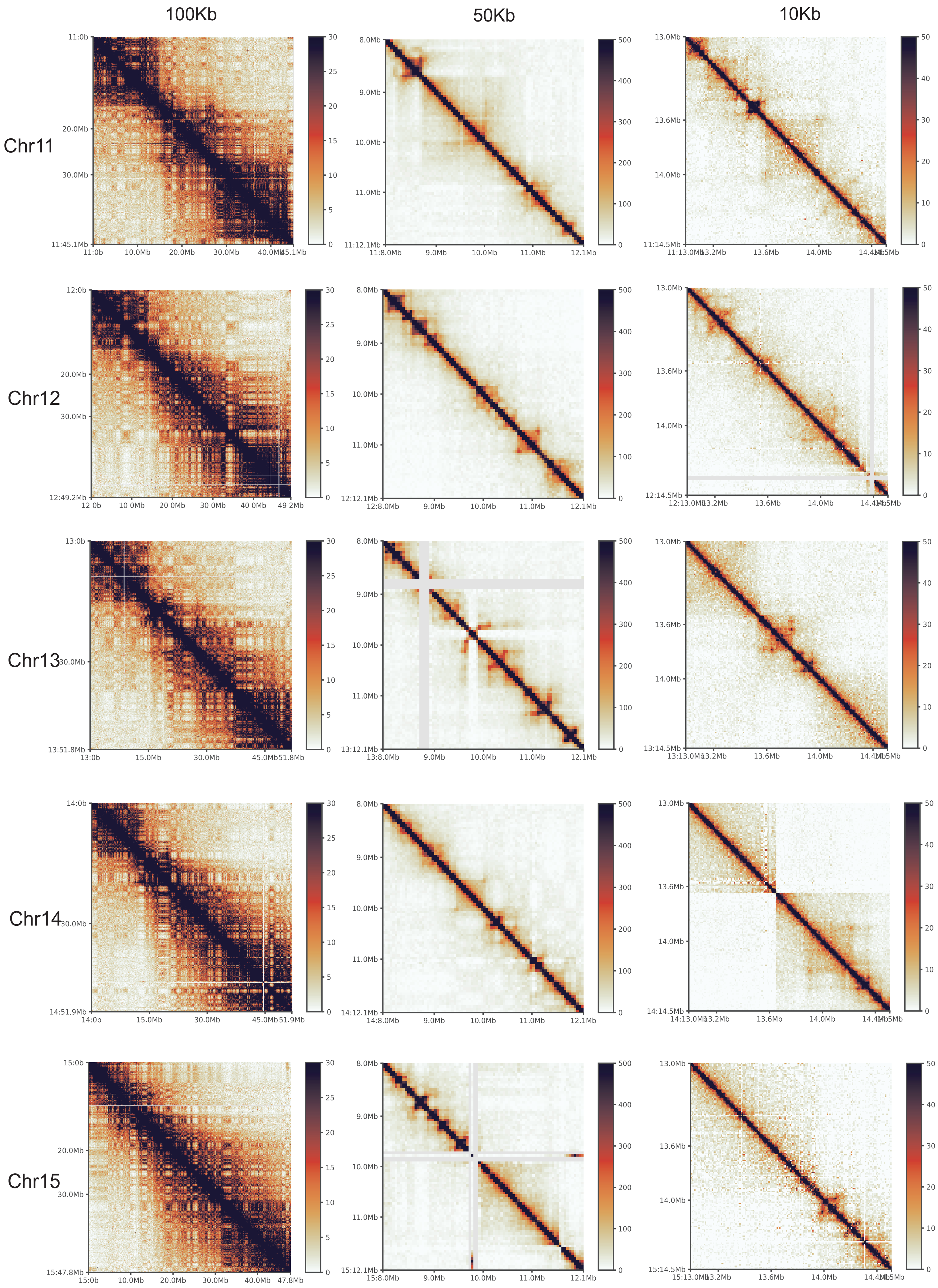
100Kb
50Kb
10Kb
Chr11
Chr12
Chr13
Chr14
Chr15
11:0b
20.0Mb
30.0Mb
11:45.1Mb
10.0Mb
40.0Mb
45.1Mb
8.0Mb
9.0Mb
10.0Mb
11.0Mb
11:12.1Mb
11:8.0Mb
12.1Mb
13.0Mb
13.6Mb
14.0Mb
11:14.5Mb
11:13.0Mb
13.2Mb
14.5Mb
12:0b
12:49.2Mb
12:12.1Mb
12:8.0Mb
12:14.5Mb
12:13.0Mb
13:0b
13:51.8Mb
15.0Mb
45.0Mb
51.8Mb
13:12.1Mb
13:8.0Mb
13:14.5Mb
13:13.0Mb
14:0b
14:51.9Mb
51.9Mb
14:12.1Mb
14:8.0Mb
14:14.5Mb
14:13.0Mb
15:0b
15:47.8Mb
47.8Mb
15:12.1Mb
15:8.0Mb
15:14.5Mb
15:13.0Mb
0
5
10
15
20
25
30
100
200
300
400
500
40
50

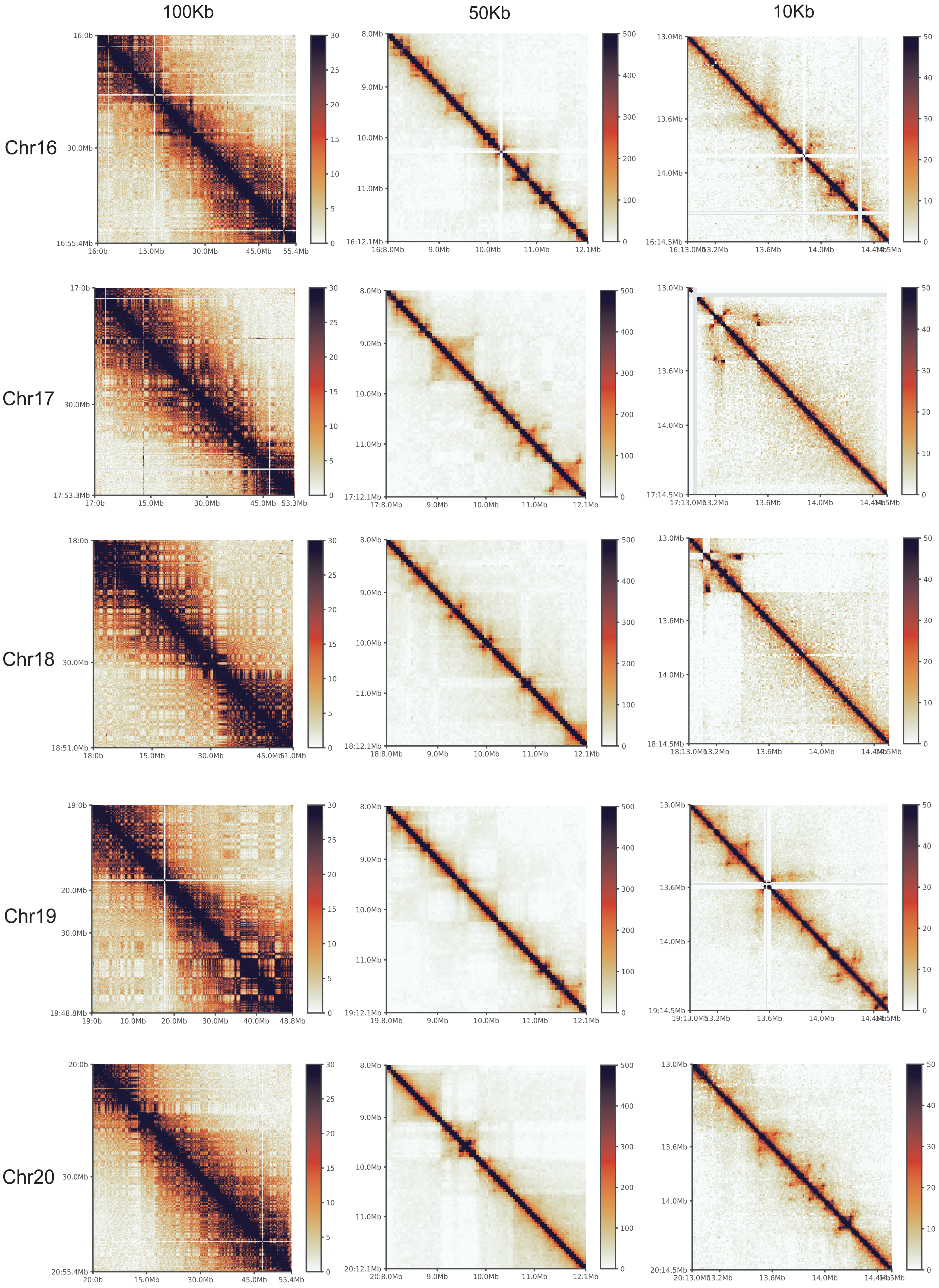
100Kb
50Kb
10Kb
Chr16
Chr17
Chr18
Chr19
Chr20

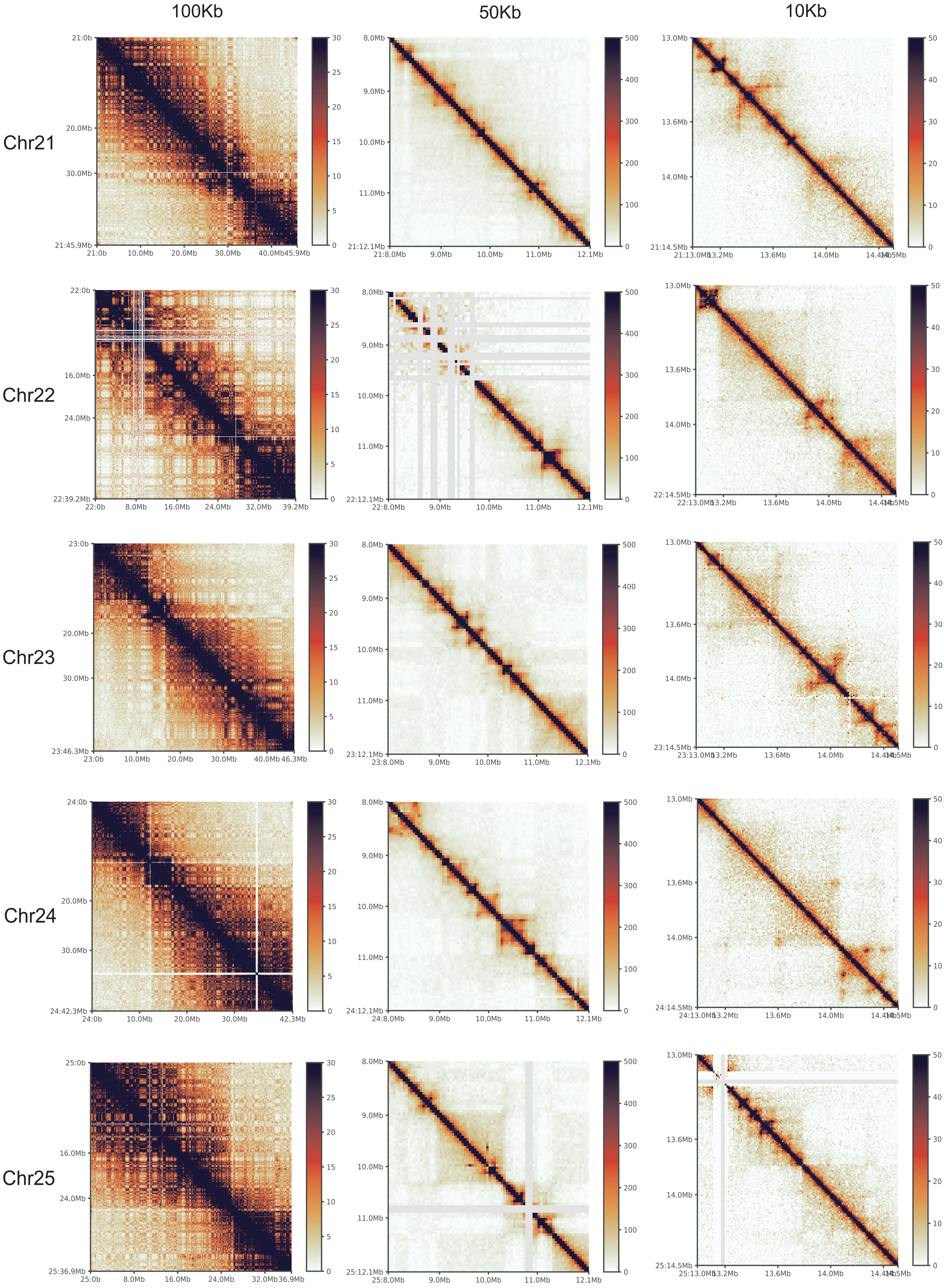

100Kb
50Kb
10Kb
Chr21
Chr22
Chr23
Chr24
Chr25
21:0b
20.0Mb
30.0Mb
21:45.9Mb
10.0Mb
40.0Mb45.9Mb
8.0Mb
9.0Mb
10.0Mb
11.0Mb
21:12.1Mb
21:8.0Mb
12.1Mb
13.0Mb
13.6Mb
14.0Mb
21:14.5Mb
21:13.0Mb13.2Mb
14.4Mb.5Mb
22:0b
16.0Mb
24.0Mb
22:39.2Mb
32.0Mb
39.2Mb
22:12.1Mb
22:8.0Mb
22:14.5Mb
22:13.0Mb13.2Mb
23:0b
23:46.3Mb
40.0Mb 46.3Mb
23:12.1Mb
23:8.0Mb
23:14.5Mb
23:13.0Mb13.2Mb
24:0b
24:42.3Mb
42.3Mb
24:12.1Mb
24:8.0Mb
24:14.5Mb
24:13.0Mb13.2Mb
25:0b
25:36.9Mb
32.0Mb36.9Mb
25:12.1Mb
25:8.0Mb
25:14.5Mb
25:13.0Mb13.2Mb
0
5
10
15
20
25
30
100
200
300
400
500
40
50
